# Somatostatin Receptor Subtype Expression in Patients with Acromegaly and Complicated Clinical Course

**DOI:** 10.3390/diagnostics11061050

**Published:** 2021-06-07

**Authors:** Robert Pichler, Ognian Kalev, Berndt Tomancok, Michael Sonnberger, Daniela Ehrlich, Marina Hodolic

**Affiliations:** 1 Institute of Nuclear Medicine, Neuromed Campus, Kepler University Hospital, 4021 Linz, Austria; robert.pichler@kepleruniklinikum.at; 2 Institute of Pathology and Neuropathology, Neuromed Campus, Kepler University Hospital, 4021 Linz, Austria; ognian.kalev@kepleruniklinikum.at; 3 Department of Neurosurgery, Neuromed Campus, Kepler University Hospital, 4021 Linz, Austria; berndt.tomancok@kepleruniklinikum.at; 4 Institute of Neuroradiology, Neuromed Campus, Kepler University Hospital, 4021 Linz, Austria; michael.sonnberger@kepleruniklinikum.at; 5 Department of Neurology, Neuromed Campus, Kepler University Hospital, 4021 Linz, Austria; daniela.ehrlich@kepleruniklinikum.at; 6 Nuclear Medicine Research Department, IASON, A-8054 Graz, Austria; 7 Nuclear Medicine Department, Faculty of Medicine and Dentistry, Palacký University Olomouc, 77900 Olomouc, Czech Republic

**Keywords:** acromegaly, somatostatin receptor subtype, immunohistochemistry, somatostatin analogues

## Abstract

Somatostatin analogues are considered to be the first line of treatment in acromegaly. Somatostatin analogues of the first generation mainly target the somatostatin receptor (SSTR) subtype 2 and have been proven efficient in the majority of patients with acromegaly. Pasireotide was the first somatostatin analogue also substantially targeting the SSTR subtype 5. An efficient drug for Cushing’s disease tailored to suboptimal-responding patients with acromegaly then became available. We immunohistochemically investigated SSTR subtypes expression in pituitary adenomas from operated acromegaly patients with clinical relapse and a complicated clinical course. Patients received pasireotide in the course of their disease. The predictive value of SSTR subtypes immunhistochemical analysis for the therapeutic response is discussed.

## 1. Introduction

Somatostatin analogues (SA) are a cornerstone in the treatment of acromegaly that have enabled efficient medical treatment since their introduction. SA of the first generation, mainly targeting the somatostatin receptor (SSTR) subtype 2, has proven to be efficient in most patients with acromegaly but not in patients with Cushing’s disease. With the development of SA targeting the SSTR subtype 5 additionally to the subtype 2, i.e., pasireotide, an efficient drug for ACTH secreting pituitary tumor patients became available [1]. Additionally, patients with acromegaly who could not be managed satisfactorily with sandostatin or lanreotide could reach endocrine control under pasireotide treatment [2].

It is feasible to investigate specific subtype-receptor density on biopsy specimens using immunohistochemistry methods; but it is not yet established to measure SSTR subtype 2 and 5 on pituitary tumour tissue to predict therapeutic response. It has been reported that high SSTR2 levels and SSTR2/SSTR5 ratio are associated with responsiveness to first generation somatostatin analogues—if not, pasireotide efficacy is favoured [3].

## 2. Materials and Methods

We investigated immunohistochemically SSTR subtypes 2 and 5 in pituitary adenomas in operated acromegaly patients with clinical relapse under treatment with SA of the first generation.

Formalin-fixed, paraffin-embedded tumor samples were cut into sequential 4-μm-thick sections, deparaffinized, and immunostained using SSTR2 (Somatostatin receptor type 2 RabMAb, Clone UMB1, Epitomics, dilution 1:300) and SSTR5 (Anti-Somatostatin Receptor 5 RabMAb, Clone UMB4, Abcam, dilution 1:100) primary antibodies. BenchMark ULTRA immunostainer with DAB visualization was used for both reactions.

SSTR2 and SSTR5 immunostaining was scored by a semiquantitative immunoreactivity scoring system and is the product of the percentage of positive-stained cells (0: no positive cells; 1: <10%; 2: 10–50%; 3: 51–80%; 4: >80%) multiplied by the staining intensity (0: no staining; 1: weak staining; 2: moderate staining; 3: strong staining). The Immuno Reactivity Score (IRS) ranges between 0 and 12 [4,5]. 

Primary Dako-antibodies against hGH (Somatotropin, Rabbit polyclonal, dilution 1:2000), AE1/AE3, and ACTH (both Abs mouse, monoclonal, dilution 1:50) further processed in Thermo Scientific autostainer 480S with DAB visualization were also used.

## 3. Results

### 3.1. Clinical Story #1

**Figure 1:**
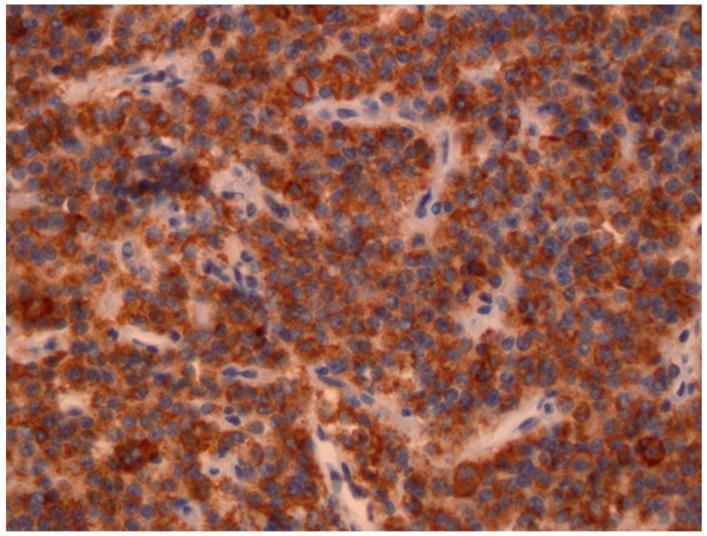
Immunohistochemical staining for growth hormone was positive in nearly all cells of the adenoma. A dense, granulated pattern was observed in this specimen (patient 1).

**Figure 2:**
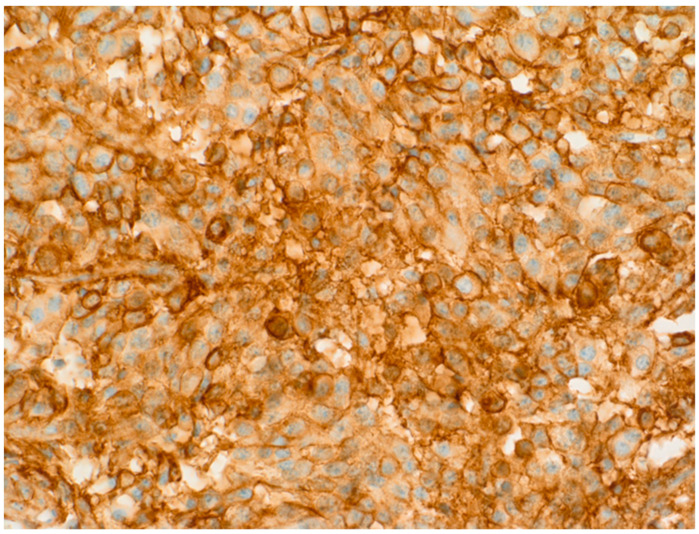
SSTR subtype 2 is present in >80% of the cells with moderate intensity of level 2; this leads to an immunoreactivity score (IRS) of 8 (patient 1).

**Figure 3:**
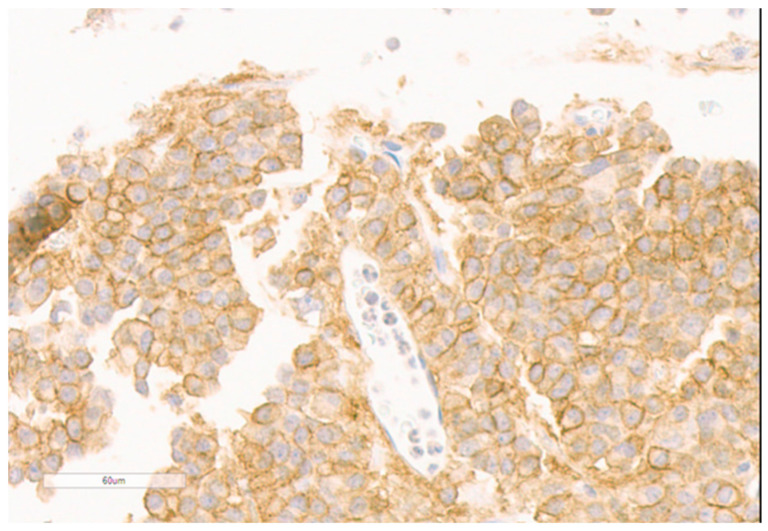
SSTR subtype 5 can be observed in about 70% of the cells at an intensity of level 2; IRS is therefore 6 (patient 1).

A 22-year-old woman presented in 2011 at our hospital for endocrine evaluation with subtle coarsening/masculinisation of facial features. The insulin-like growth factor 1 (IGF-1) was elevated to two times the upper limit of normal (ULN), and the oral glucose tolerance test could not show growth hormone (GH) suppression. Laboratory values are presented by Table 1. All other hormonal values were normal. MRI detected a pituitary adenoma with a diameter of 12 mm (T2 signal was isointensive), which was operated via transsphenoidal approach in August 2011. The densely granulated somatotroph adenoma stained positive for GH (Figure 1), and Ki-67 reached 5% focally. Because of endocrine relapse. 2012 lanreotide 60 mg/month was started; the only clinical complaint was facial skin acne. Later, cabergoline 1 mg/week was added, but in spite of combined medical therapy, MRI control revealed an intrasellar tumor (14 mm) in May 2013. The endocrine situation remained favourable. Reoperation showed the same histology as before. No medical therapy was given then. In 2015, an endocrine relapse with modest elevation of IGF-1 levels occurred, and a growing residual tumor mass (the diameter was 9 mm then) became detectable by MRI. Higher dose cabergoline therapy was reinitiated without success. Stereotactic radiation with 18 Gy was performed in January 2016. Laboratory parameters of the GH-axis normalized after some weeks without any further therapy. In November 2016, IGF-1 levels rose again to 1.2 ULN and skin acne became worse. In the meantime, pasireotide therapy had become available in Austria for acromegaly patients and was initiated at low dose (20 mg/month) in 2017. Then SSTR subtype evaluation of the preserved tumor specimens was additionally performed (Figure 2 and Figure 3). Since then, all further control examinations until September 2020 presented normal IGF-1 values and GH < 1.5 ng/mL. The patient did not refer any clinical problems. Adverse events such as diabetes mellitus did not occur. Control MRI presented size regression of the pituitary tumour to 2 mm at last control (see Table 1).

### 3.2. Clinical Story #2

**Figure 4:**
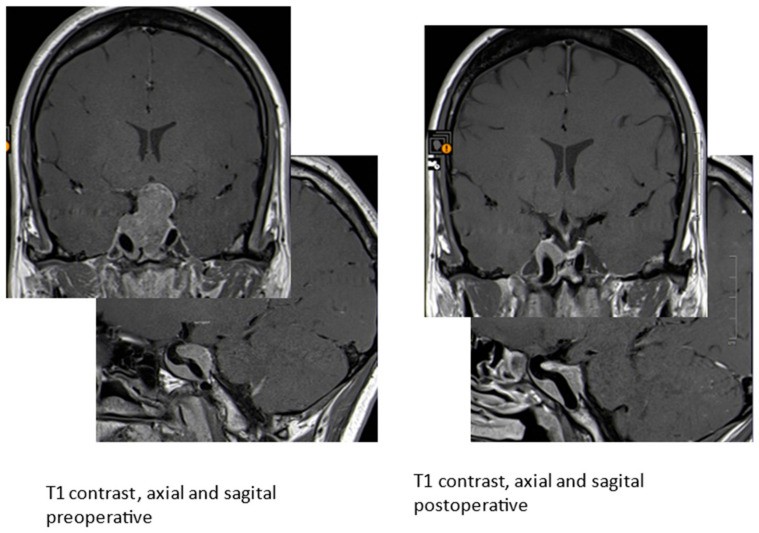
MRI of the sella T1 with contrast media, axial and sagital pre- and postoperative: the tumor encloses the carotid arteries; close to the arteries, remnants had to be left (patient 2).

**Figure 5:**
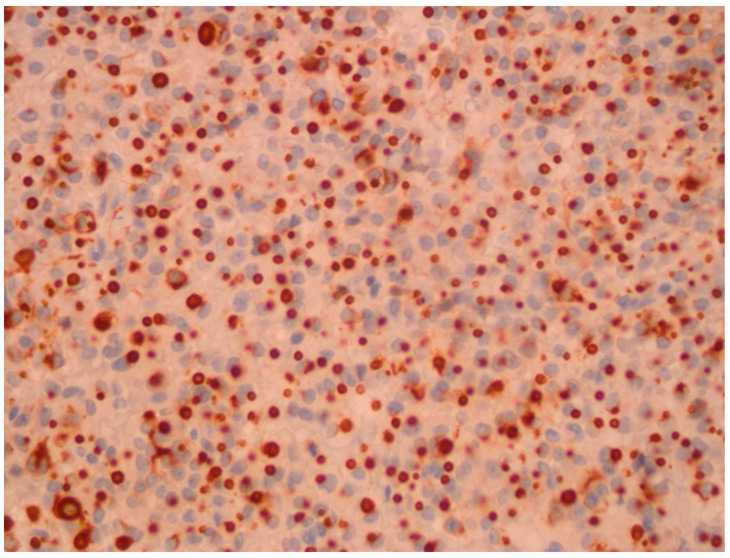
Abundant fibrous bodies were present in the pituitary adenoma species (patient 2).

**Figure 6:**
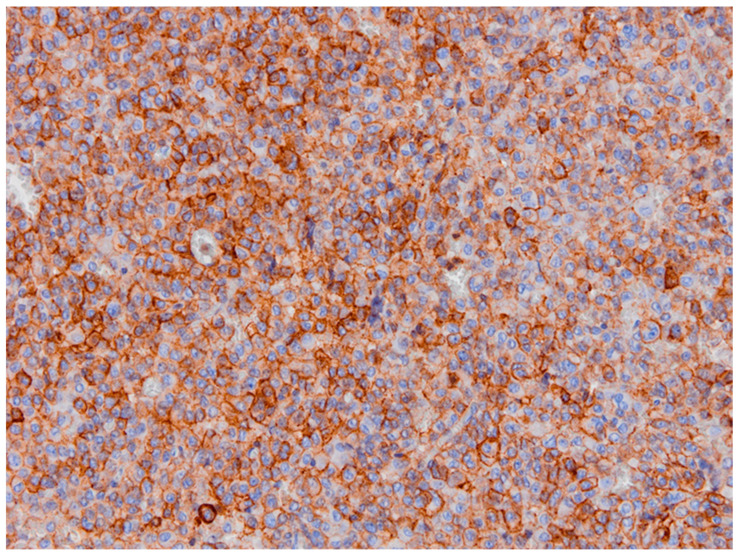
At moderate staining intensity of SSTR, subtype 2 and IRS of 6 can be observed (patient 2).

**Figure 7:**
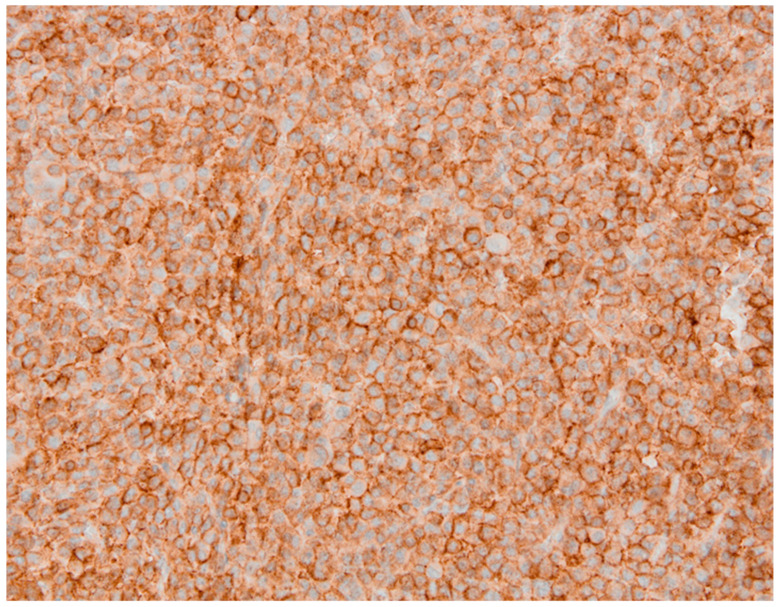
For SSTR5—presented here—the IRS of 8 is higher than that for SSTR2 (patient 2).

A 32-year-old female patient presented at the department of neurology with visual field restrictions in June 2016 that were caused by a large pituitary tumor with a maximal diameter of 37 mm, presenting with mixed hypo- and isointensive signal on T2 images by MRI. Medical history revealed carpal tunnel syndrome and nodular goiter. MRI had been effectuated 10 years before because of tinnitus without any signs of a hypophyseal lesion. There was some evidence of enlargement of the fingers over the years suggested by rings (which became too small and did not fit any more) and an increasing size of shoes was inquired. Mild galactorrhea was present due to concomitant hyperprolactinemia. HGH levels were elevated, and IGF-1 preoperatively reached 2.7 × ULN. Laboratory values are presented in Table 2. Morning ACTH (51 pg/mL) and serum cortisol (26.4 µg/dl) were in the upper normal range; free cortisol in 24 h urine sample (52 µg/d) was in the normal range of 36–137 µg/d. Prolactin was also normal at 19.6 ng/mL. As the carotid arteries were enclosed by the tumor (Figure 4), radiotherapy was principally considered after transsphenoidal surgery because of expected tumor remnants. Immunohistochemistry was positive for HGH only; therefore, hyperprolactinemia was considered to be caused by a hampered feedback mechanism of the hypothalamic-pituitary axis [6]. Ki-67 focally reached 5% (Table 3). Abundant fibrous bodies were present (Figure 5), corresponding to a sparsely granulated somatotroph adenoma with expected limited therapeutic efficacy. The endocrine response to surgery was poor, as IGF-1 remained relatively stable at 2.5 UNV. As lanreotide 40 mg/month—the recommended starter dose at the time in Austria—diminished, IGF-1 only at 2.0 × ULN therapy was changed to pasireotide 40 mg/month. This worked better, and a IGF-1 level of 1.5 × ULN was obtained. The addition of cabergoline was without any benefit and was therefore stopped. LINAC radiation surgery was then realized in November 2017 with 18 Gy, pasireotide being continued as a bridging therapy. Six months later, IGF-1 was even higher at 2.0 × ULN, and pegvisomant was added. MRI showed the tumor remnants with identical size. Finally, the patient had an IGF-1 level of 1.25 ULN under pasireotide and a then augmented therapy of pegvisomant 60 mg/week in March 2019. Pegvisomant was decided to be increased at 80 mg/week and further augmented to 100 mg/week and to 150 mg/week in April 2020 and to 180 mg/week after October 2020. Clinically, she reported some mild arthropathy, which ceased in October 2020 when IGF-1 had nearly normalized; otherwise, she felt well. Drug-induced, adverse events were not present; HbA1c and liver parameters were in the normal range. Retrospectively, SSTR subtype analysis of the adenoma was realized (Figure 6 and Figure 7).

## 4. Discussion

Long-acting somatostatin analogs of the first generation had become a cornerstone of medical therapy for acromegaly [7]. As they target mainly the SSTR subtype 2, they were not useful for treating Cushing’s disease. The field changed with the development of pasireotide [8]. This new generation somatostatin analog also became available for treatment of acromegaly and has proven to be superior in various cases but at the price of a higher rate of adverse effects, especially diabetes mellitus [9,10]. It would be desirable to have a diagnostic marker to know in advance if the use of pasireotide is preferable to first-generation somatostatin analogs. The presence or density of the SSTR subtypes shown by immunohistochemistry of the tumor specimens might be such an option.

Patients presented here received pasireotide in the course of their disease. Our two cases showed a pronounced receptor density of subtype 5. Regardless, the therapeutic change was decided based on endocrine response (hormone levels and clinics) only. Immunohistochemistry for SSTR subtypes was done retrospectively on the preserved tumor species. 

Higher SSTR2 expression on somatotroph adenomas is considered to generate a better response to somatostatin analogues [4]. It has to be emphasized that the responsiveness to pasireotide has been shown to depend on the presence of receptor subtype 2 [11] but also that SSTR5 might be the major determinant of the biochemical response [12]. It can be speculated that a low intensity of subtype 2 might be a reason to switch early to alternatives and/or combination therapies with pegvisomant [13] and cabergoline [14]. In our cases, cabergoline was been efficient. Other experience shows an if not disappointing then at least suboptimal therapeutic response to pasireotide, probably due to only moderate expression of SSTR subtype 2. As SSTR subtype 5 expression outnumbered that of subtype 2, the slightly better efficiency of pasireotide compared to lanreotide could be explained by this circumstance. We also relate the relatively unfavorable evolution of tumor growth in case 1 and, respectively, endocrine response to radiation therapy in case 2 to the relatively high Ki67-labeling indices of the adenomas [15]. 

Receptor profiling and possibly the molecular characterization of pituitary tumors may guide an individualized therapeutic plan [16].

## 5. Conclusions

We recommend the evaluation of SSTR subtypes 2 and 5 in pituitary adenomas of patients with acromegaly in prospective studies to define the usefulness for early therapeutic decisions regarding somatostatin analogues. 

## Figures and Tables

**Table 1:** Patient 1 laboratory data.

Date	GH (ng/mL)	IGF-1 (ng/mL)	Normal Value IGF-1	Therapy (mg)	MRI (mm) Length/Width/Height
August 2011	10.9	563	117–358	0	14/12/12
August 2011				1st operation	8/11/7 **
September 2011	4.9	352	117–358	0	
December 2011	10.6	334	117–358	0	
June 2012	10.6	334	117–358	LAN90/8w	
December 2012	6.1	752	117–358	LAN120/6w	10/14/11
March 2013	9.8	504	117–358	LAN90/4w+CAB1/1w	
May 2013					10/14/11
June 2013	9.9	438	117–358	LAN90/4w+CAB4/1w	
October 2013	18.6	412	117–358	LAN90/4w+CAB6/1w	11/15/11
November 2013	3.0	312	117–358	2nd operation	2/2/2
December 2013	1.8	322	117–358	0	
March 2014	10.2	164	117–358	0	2/2/2
July 2014	3.5	190	117–358	0	
January 2015	4.1	292	117–358	0	5/6/6
January 2016	8.7	568	117–358	CAB1/1w	9/9/7
January 2016				Radiation	9/9/7
May 2016	2.2	381	117–358	0	6/6/5
August 2016	2.6	328	117–358	0	
November 2016	3.4	401	117–358	0	
February 2017	1.3	228150	117–358 88–537 *	PAS20/4w	
June 2017	1.8	189145	117–358 88–537 *	PAS20/4w	2/2/2
January 2018	1.2	133	88–537	PAS20/4w	
June 2018	1.4	149	88–537	PAS20/4w	2/2/2
November 2018	1.2	209	41–246	PAS20/4w	
August 2019	1.0	170	112–281	PAS20/4w	2/2/2
September 2020	1.2	152	109–271	PAS20/4w	

* new laboratory kit, parallel measurement, ** intraoperative MRI, LAN, lanreotide; CAB, cabergoline; PAS, pasireotide.

**Table 2:** Patient 2 laboratory data.

Date	GH (ng/mL)	IGF-1 (ng/mL)	Normal Value IGF-1	Therapy (mg)	MRI (mm) Length/Width/Height
June 2016	>40	816	109–307	0	26/25/37
June 2016			109–307	Operation	
June 2016	5.0	751	109–307	0	8/15/11
October 2016	8.5	760	109–307	0	
February 2017	10.6	678 400	109–307 41–246 *	LAN60/4w	
March 2017	11.5	604 405	109–307 41–246 *	LAN60/4w	
May 2017	6.4	603 360	109–307 41–246 *	PAS40/4w	8/15/11
August 2017	3.9	581 373	109–307 41–246 *	PAS40/4w+CAB1/1w	
November 2017	6.0	425	41–246	PAS40/4w	8/15/11
November 2017				Radiation	
May 2018	5.9	490	41–246	PAS40/6w	8/15/11
September 2018	2.3	287	41–246	PAS40/6w+PEG40/1w	
March 2019	11.8	304	100–242	PAS40/6w+PEG60/1w	
August 2019	7.2	297	98–238	PAS40/6w+PEG80/1w	6/10/9
April 2020	8.6	319	98–238	PAS40/6w+PEG100/1w	
October 2020	14.9	246	97–234	PAS40/6w+PEG150/1w	
May 2021	13,0	138	97–234	PAS40/6w+PEG180/1w	

* new laboratory kit, parallel measurement; PEG, pegvisomant.

**Table 3:** Prognostic factors.

	Patient 1	Patient 2
Adenoma size	Macroadenoma (12 mm)	Macroadenoma (37 mm)
Histology	Densely granulated	Sparsely granulated, fibrous bodies
Ki67	5%	5%
SSTR2	IRS = 8	IRS = 6
SSTR5	IRS = 6	IRS = 8

## Data Availability

Patients were under the clinical routine work; ethical committee approval was obtained.

## References

[1] Boscaro M., Ludlam W.H., Atkinson B., Glusman J.E., Petersenn S., Reincke M., Snyder P., Tabarin A., Biller B.M., Findling J. (2009). Treatment of pituitary-dependent Cushing’s disease with the multireceptor ligand somatostatin analog pasireotide (SOM230): A multicenter, phase II trial. J. Clin. Endocrinol. Metab..

[2] Paragliola R.M., Salvatori R. (2018). Novel Somatostatin Receptor Ligands Therapies for Acromegaly. Front. Endocrinol..

[3] Marazuela M., Ramos-Leví A.M., Borges de Souza P., Zatelli M.C. (2018). Is receptor profiling useful for predicting pituitary therapy?. Eur. J. Endocrinol..

[4] Franck S.E., Gatto F., van der Lely A.J., Janssen J.A.M.J.L., Dallenga A.H.G., Nagtegaal A.P., Hofland L.J., Neggers S.J.C.M.M. (2017). Somatostatin Receptor Expression in GH-Secreting Pituitary Adenomas Treated with Long-Acting Somatostatin Analogues in Combination with Pegvisomant. Neuroendocrinology.

[5] Remmele W., Stegner H.E. (1987). Recommendation for uniform definition of an immunoreactive score (IRS) for immunohistochemical estrogen receptor detection (ER-ICA) in breast cancer tissue (in German). Pathologe.

[6] Mikhael S., Punjala-Patel A., Gavrilova-Jordan L. (2019). Hypothalamic-Pituitary-Ovarian Axis Disorders Impacting Female Fertility. Biomedicines.

[7] Gadelha M.R., Kasuki L., Korbonits M. (2013). Novel pathway for somatostatin analogs in patients with acromegaly. Trends Endocrinol. Metab..

[8] Colao A., Bronstein M.D., Freda P., Gu F., Shen C.C., Gadelha M., Fleseriu M., van der Lely A.J., Farrall A.J., Hermosillo Reséndiz K. (2014). Pasireotide versus octreotide in acromegaly: A head-to-head superiority study. J. Clin. Endocrinol. Metab..

[9] Sheppard M., Bronstein M.D., Freda P., Serri O., De Marinis L., Naves L., Rozhinskaya L., Hermosillo Reséndiz K., Ruffin M., Chen Y. (2015). Pasireotide LAR maintains inhibition of GH and IGF-1 in patients with acromegaly for up to 25 months: Results from the blinded extension phase of a randomized, double-blind, multicenter, Phase III study. Pituitary.

[10] Gadelha M.R., Bronstein M.D., Brue T., Coculescu M., Fleseriu M., Guitelman M., Pronin V., Raverot G., Shimon I., Lievre K.K. (2014). Pasireotide versus continued treatment with octreotide or lanreotide in patients with inadequately controlled acromegaly (PAOLA): A randomised, phase 3 trial. Lancet Diabetes Endocrinol..

[11] Muhammad A., Coopmans E.C., Gatto F., Franck S.E., Janssen J.A.M.J.L., van der Lely A.J., Hofland L.J., Neggers S.J.C.M.M. (2019). Pasireotide Responsiveness in Acromegaly Is Mainly Driven by Somatostatin Receptor Subtype 2 Expression. J. Clin. Endocrinol. Metab..

[12] Iacovazzo D., Carlsen E., Lugli F., Chiloiro S., Piacentini S., Bianchi A., Giampietro A., Mormando M., Clear A.J., Doglietto F. (2016). Factors predicting pasireotide responsiveness in somatotroph pituitary adenomas resistant to first-generation somatostatin analogues: An immunohistochemical study. Eur. J. Endocrinol..

[13] van der Lely A.J., Biller B.M., Brue T., Buchfelder M., Ghigo E., Gomez R., Hey-Hadavi J., Lundgren F., Rajicic N., Strasburger C.J. (2012). Long-term safety of pegvisomant in patients with acromegaly: Comprehensive review of 1288 subjects in ACROSTUDY. J. Clin. Endocrinol. Metab..

[14] Sandret L., Maison P., Chanson P. (2011). Place of cabergoline in acromegaly: A meta-analysis. J. Clin. Endocrinol. Metab..

[15] Dai C., Liu X., Ma W., Wang R. (2019). The Treatment of Refractory Pituitary Adenomas. Front. Endocrinol..

[16] Alexandraki K.I., Papadimitriou E., Mavroeidi V., Kyriakopoulos G., Xydakis A., Papaioannou T.G., Kolomodi D., Kaltsas G.A., Grossman A.B. (2019). Role of Receptor Profiling for Personalized Therapy in a Patient with a Growth Hormone-Secreting Macroadenoma Resistant to First-Generation Somatostatin Analogues. J. Pers. Med..

